# Expression Profile of Genes Potentially Associated with Adequate Glycemic Control in Patients with Type 2 Diabetes Mellitus

**DOI:** 10.1155/2017/2180819

**Published:** 2017-07-25

**Authors:** Sâmia Cruz Tfaile Corbi, Alliny Souza Bastos, Rafael Nepomuceno, Thamiris Cirelli, Raquel Alves dos Santos, Catarina Satie Takahashi, Cristiane S. Rocha, Silvana Regina Perez Orrico, Claudia V. Maurer-Morelli, Raquel Mantuaneli Scarel-Caminaga

**Affiliations:** ^1^Department of Diagnosis and Surgery, School of Dentistry, Universidade Estadual Paulista (UNESP), Araraquara, SP, Brazil; ^2^Department of Morphology, School of Dentistry, Universidade Estadual Paulista (UNESP), Araraquara, SP, Brazil; ^3^Postgraduate Program in Sciences of the University of Franca, Franca, SP, Brazil; ^4^Department of Genetics, Faculty of Medicine of Ribeirão Preto and Department of Biology, FFCLRP, University of São Paulo (USP), Ribeirão Preto, SP, Brazil; ^5^Department of Medical Genetics, University of Campinas (UNICAMP), Campinas, SP, Brazil

## Abstract

Despite increasing research in type 2 diabetes mellitus (T2D), there are few studies showing the impact of the poor glycemic control on biological processes occurring in T2D. In order to identify potential genes related to poorly/well-controlled patients with T2D, our strategy of investigation included a primary screen by microarray (Human Genome U133) in a small group of individuals followed by an independent validation in a greater group using RT-qPCR. Ninety patients were divided as follows: poorly controlled T2D (G1), well-controlled T2D (G2), and normoglycemic individuals (G3). After using affy package in R, differentially expressed genes (DEGs) were prospected as candidate genes potentially relevant for the glycemic control in T2D patients. After validation by RT-qPCR, the obtained DEGs were as follows—G1 + G2 versus G3: *HLA-DQA1*, *SOS1*, and *BRCA2*; G2 versus G1: *ENO2*, *VAMP2*, *CCND3*, *CEBPD*, *LGALS12*, *AGBL5*, *MAP2K5*, and *PPAP2B*; G2 versus G3: *HLA-DQB1*, *MCM4*, and *SEC13*; and G1 versus G3: *PPIC*. This demonstrated a systemic exacerbation of the gene expression related to immune response in T2D patients. Moreover, genes related to lipid metabolisms and DNA replication/repair were influenced by the glycemic control. In conclusion, this study pointed out candidate genes potentially associated with adequate glycemic control in T2D patients, contributing to the knowledge of how the glycemic control could systemically influence gene expression.

## 1. Introduction

Diabetes mellitus (DM), characterized by hyperglycemia, is associated with the progressive development of complications, like atherosclerosis, renal and neuronal damage, and blindness [1]. Type 2 diabetes mellitus (T2D), the most common form of diabetes mellitus (DM) accounting for 80–90% of the cases, is a chronic polygenic disorder identified by defects in insulin action and/or deficiencies in pancreatic insulin secretion [2, 3]. It is known that T2D occurs concomitantly to other systemic diseases, such as dyslipidemia [4] and cardiovascular diseases [5]. Patients affected by these diseases have demonstrated a hyperinflammatory state [6–8] that put their health at major risk [9, 10]. It has been shown that chronically elevated levels of inflammatory markers in T2D patients with poor glycemic control can increase the risk for myocardial infarction [11] and infectious diseases, including chronic periodontitis [12].

Adequate glycemic control remains a challenge for clinicians; thus, more studies are needed to investigate the impact of poor glycemic control on diverse biological mechanistic processes in patients with T2D. It is already known that hyperglycemia can lead to vascular complications via several mechanisms, and clinical trials have reported that intensive glycemic control significantly reduces microvascular complications, for instance, retinopathy, nephropathy, and peripheral arterial disease [13–15].

A significant difference in the expression of genes involved in insulin signaling and immune response, for example, TNF*α*, interleukin- (IL-) 6, and IL-1*β*, was found in PBMCs from T2DM and impaired glucose tolerance patients [16–18]. The influence of hyperglycemia on gene expression, somewhat, can explain the distinct expression profiles displayed by diabetic patients compared to the control group, such as fatty acid metabolism and protection against lipid-induced oxidative stress, adipocytokine, and ceramide signaling pathways [16].

Despite previous reports, adequate glycemic control remains a challenge for clinicians; thus, additional studies are necessary to investigate the impact of poor glycemic control on patients with T2D. Therefore, we utilized a primary microarray screening to prospect candidate genes potentially relevant for the glycemic control in T2D patients, and to validate this finding, we investigated their expression levels in T2D patients with poor or good glycemic control and in normoglycemic individuals.

## 2. Materials and Methods

### 2.1. Study Population and Physical and Biochemical Evaluations

This study was approved by the Ethics in Human Research Committee of School of Dentistry (Universidade Estadual Paulista (UNESP), Araraquara, Brazil; Protocol number 50/06) and was conducted according to the ethical principles of the Declaration of Helsinki. During three years (2009–2011), after we examined 1788 patients, we selected 90 patients according to our criteria of inclusion/exclusion as detailed in our previous studies [19–21]. From the selected patients, blood samples were collected after a 12-hour overnight fast for the evaluation of fasting plasma glucose (mg/dl), glycated hemoglobin (HbA1c), and insulin levels (U/l), in which all the analyses were performed by the same laboratory. Physical examinations including measurement of waist and hip circumference (centimeters), height (meters), weight (kilograms), and body mass index (BMI) were evaluated in all selected patients.

Based upon diabetic and glycemic control statuses, monitored by an endocrinologist, 90 patients were enrolled in this study who were divided into three groups (G) of 30 patients each as follows: poorly controlled T2D (G1, HbA1c ≥ 8.5%), well-controlled T2D (G2, HbA1c < 7.0%), and normoglycemic individuals (G3, HbA1c < 6.5%) [19]. Patients from G1, G2, and G3 were tightly matched for the presence of two common comorbidities: dyslipidemia and chronic periodontitis, as previously demonstrated in Bastos et al. [20] and Corbi et al. [21].

### 2.2. Selection of Candidate Genes

We used a list of differentially expressed genes (DEGs) based on microarray analysis (Human U133 Plus 2.0 Affymetrix Inc., Santa Clara, CA, USA—unpublished data) to select candidate genes in poorly and well-controlled T2D as well as normoglycemic patients. Microarray data was generated from patients of G1 (*n* = 5), G2 (*n* = 7), and G3 (*n* = 6), after considering greater homogeneity regarding biochemical parameters. DEGs were selected as follows: G1 + G2 versus G3, in order to compare T2D patients (independent of glycemic control) with normoglycemic individuals; G2 versus G1, to access the effect of good glycemic control on the gene expression, in comparison with poorly controlled T2D patients; G2 versus G3, to access the effect of good glycemic control on the T2D patients in comparison with normoglycemic individuals; and G1 versus G3, to access the effect of poor glycemic control on the T2D patients in comparison with normoglycemic individuals.

### 2.3. Reverse Transcription-Quantitative Polymerase Chain Reaction (RT-qPCR) Real-Time Analysis

For this study, each group was composed of 30 patients (including patients chosen for microarray analysis) totalizing 90 patients. Reverse transcription reactions for the complementary DNA (cDNA) were made utilizing the SuperScript III First Strand Synthesis SuperMix (Invitrogen). Considering together the three studied groups, three genes were identified by microarray data as demonstrating the best housekeeping expression patterns: *ACTG1* (Hs03044422_g1), *RPL7A* (Hs00605223_g1), and *GAPDH* (Hs02758991_g1). The mean values of them were used as endogenous controls of the qPCR reactions. All reactions were performed in duplicate utilizing TaqMan® gene expression assays (Applied Biosystems) in the 7500 Real-Time PCR System (Applied Biosystems, Foster City, CA, USA). PCR cycling parameters were 2 minutes at 50°C, 10 minutes at 95°C and 40 cycles of 15 seconds at 95°C, and 60 seconds at 60°C. To calculate gene expression, the Expression Suite Software was used (Applied Biosystems, Foster City, CA, USA), which employs the comparative C*τ* (ΔC*τ*) method for multiplate data analysis.

### 2.4. Statistical Analysis

General characteristics of each group were described by mean and standard deviation (SD). The distribution and normality of the demographic and clinical variables were evaluated by the D'Agostino-Pearson test. Accordingly, we used the chi-squared test (for gender), and to compare other characteristics among the three groups, we used the Kruskal-Wallis test (followed by Dunn's posttest), alternatively, when only two groups were compared.

For RT-qPCR analyses, values of 2^−ΔC*τ*^ were compared between each two groups by the Mann–Whitney test using a significance level of *p* = 0.05. These analyses were carried out in the GraphPad Prism software, version 5.0.

## 3. Results

### 3.1. Sample Population

Table 1 shows demographic, physical, and biochemical data of the investigated patients. Gender, age, ethnicity, and socioeconomic status were similar among the three groups. Independent of the group, all individuals were overweight, and the diabetic patients were obese and presented higher values of BMI, abdominal circumference, and waist/hip proportions. This information can also be found in our previous studies [19–21].

### 3.2. Candidate Genes and RT-qPCR

Candidate genes selected to the RT-qPCR study are shown in Table 2. Thirty individuals were investigated for each gene, and after obtaining the Cts in the qPCR, we withdraw the outliers (mean of five individuals who presented huge difference regarding the group). We chose to use this approach in order to ensure that the significance was due the group and not influenced by a single individual.

**Figure 1:**
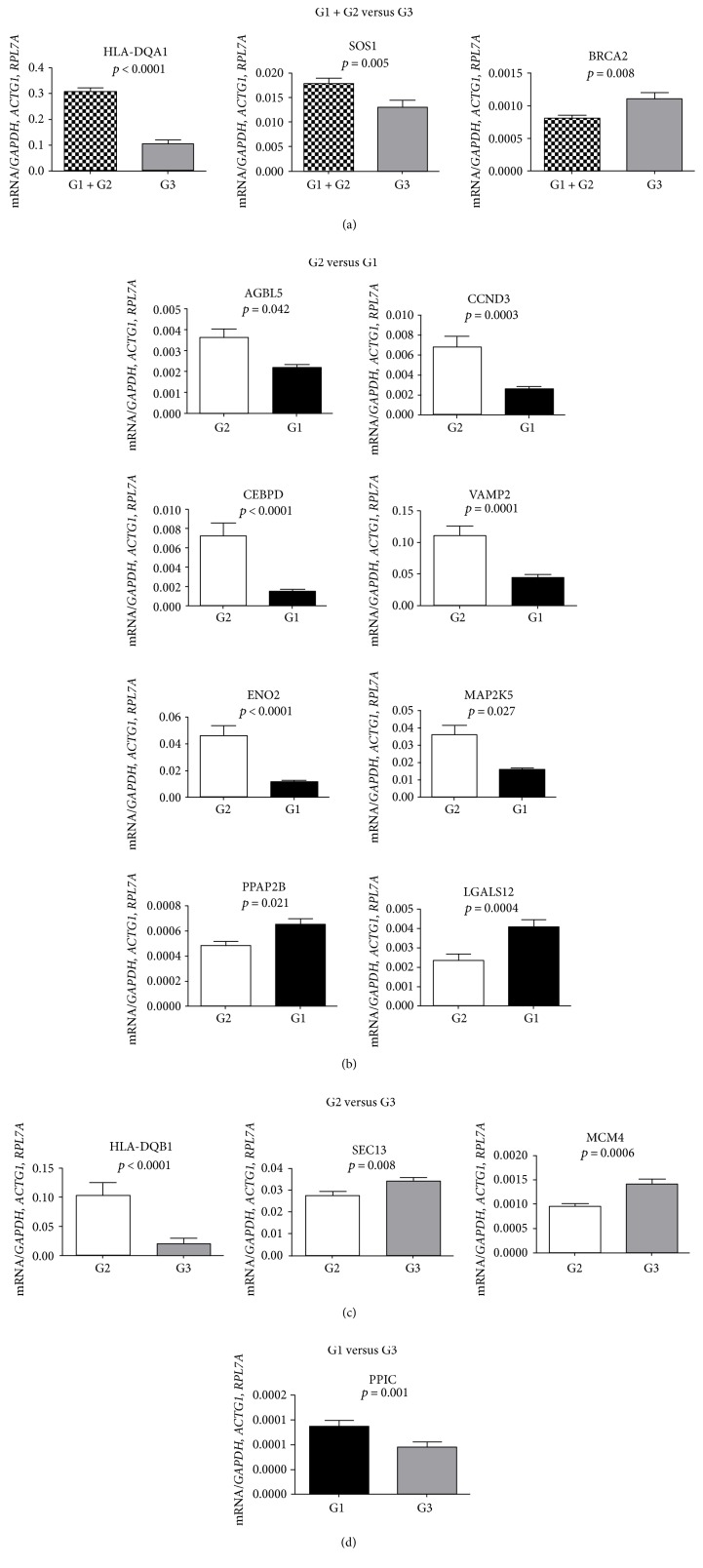
Validation results by RT-qPCR of the genes considering the different comparisons. (a) G1 + G2 versus G3; (b) G2 versus G1; (c) G1 versus G3; (d) G2 versus G3. All mRNA levels of the investigated genes were normalized to a mean of the endogenous controls *GAPDH*, *ACTG1*, and *RPL7A* genes. Data represent the mean ± SEM of 30 patients per group (Mann–Whitney *U* test; *α* = 5%). *p* values are presented in Table 2.

In the G1 + G2 versus G3 comparison, which evaluated the influence of T2D independent of glycemic control, we found that the *HLA-DQA1* (*p* < 0.0001) and *SOS1* (*p* = 0.005) genes were upregulated and the *BRCA2* (*p* = 0.008) was downregulated. The qPCR results are presented in Figure 1(a). The full name of each gene can be found in Table 2.

Regarding G2 versus G1, which evaluated the influence of the glycemic control in patients with T2D, the genes *AGBL5* (*p* = 0.043), *CCDN3* (*p* = 0.0003), *CEBPD* (*p* < 0.0001), *VAMP2* (*p* = 0.0001), *ENO2* (*p* < 0.0001), and *MAP2K5* (*p* = 0.027) were significantly upregulated in the G2 subjects compared to the G1 subjects (Figure 1(b)). In addition, the *PPAP2B* (*p* = 0.021) and *LGALS12* (*p* = 0.0004) genes were downregulated (Table 2).

Comparison between G2 versus G3, which evaluated the influence of T2D with good glycemic control, showed that the *HLA-DQA1* (*p* = <0.0001) and *HLA-DQB1* (*p* = <0.0001) genes were upregulated and the *MCM4* and *SEC13* genes were downregulated DEGs in G2 subjects (*p* = 0.0006 and *p* = 0.008, resp., Figure 1(c)). And lastly, the comparison of G1 versus G3, which evaluated the influence of poor glycemic control of T2D patients, showed that the *PPIC* gene was upregulated in the G1 (*p* = 0.001, Table 2, Figure 1(d)).

## 4. Discussion

Despite the knowledge in the field, the molecular processes regarding the adequate or poor glycemic control in patients with T2D remain poorly understood. Additional studies seeking to investigate DGEs in these conditions might shed some light on the molecular processes underlying it. In the present study, we investigated candidate genes potentially relevant for the glycemic control in a large cohort of patients with poorly or well-controlled T2D subjects and normoglycemic individuals. It is important to highlight that our cohort of patients was strictly selected after a complete clinical evaluation. The quality and robustness of the eligibility criteria certainly contributed to promote confident findings.

Candidate genes were chosen from a panel of DEGs obtained from microarray data (unpublished data). In order to identify DEGs in T2D, independent of the glycemic control, we compared G1 + G2 versus G3. Using this approach, we selected the *HLA-DQA1* and *HLA-DQB1* as candidate genes. Our results showed that both genes were upregulated in T2D patients (Figures 1(a) and 1(c)). Major histocompatibility complex (MHC), class II, is a heterodimer of molecules consisting of an alpha (DQA) and a beta chain (DQB), both anchored in the membrane. It plays a central role in the immune system by presenting peptides derived from extracellular proteins. Class II molecules are expressed in antigen-presenting cells (APC: B lymphocytes, dendritic cells, and macrophages) (http://v4.genecards.org/cgi-bin/carddisp.pl?gene=HLA-DQB1&UserNotification=BetaRedirect). *HLA-DQA1-DQB1* genes are related to leukocyte chemotaxis; G-protein signaling; IL-17-induced mucin expression; Th1, Th2, Th9, Th17, and Th22 cell differentiations; and immunological synapse formation. The current results led us to suppose that the *HLA-DQA1-DQB1* overexpression in T2D patients, mainly in well-controlled diabetics (G2), could indicate that an adequate glycemic control might reduce inflammatory response, supporting the link between inflammation and glucose metabolic disturbance [22]. Interestingly, in spite of the MHC class II molecules *HLA-DQA1* and *DQB1* being classically associated with type 1 DM, we observed their high expression in the investigated patients with T2D. A higher frequency of the high-risk *HLA-DQB1*^∗^0201/0302 was found in patients with latent autoimmune diabetes in adults (LADA) [23]. Also, a study comprised of 695 families with patients affected by type 1 and others by type 2 DM (mixed families) had HLA class II risk haplotypes, such as DR3(17)-DQA1^∗^0501-DQB1^∗^02. This result supports a possible genetic interaction between type 1 and type 2 diabetes mediated by the HLA locus [24].

Patients with T2D (G1 + G2) showed an upregulation of the *SOS1* in comparison to the normoglycemic individuals (G3). The *SOS1* gene is related to immune response pathways, such as IL-7 signaling in B lymphocytes, regulation of lipid metabolism, and the insulin signaling pathway [25]. Myers and colleagues described that during insulin stimulation, insulin receptor-expressing cells result in the tyrosine phosphorylation of SHC-transforming protein 1 or insulin receptor substrate 1 (IRS-1), which are both associated with growth factor receptor-bound protein 2 (GRB-2). GRB-2 activates p2l-RAS by forming a stable complex through its SH3 domains to SOS-1 [25]. Moreover, the *SOS1* gene is involved in the regulation of lipid metabolism and insulin signaling generic cascades (http://lsresearch.thomsonreuters.com/maps/724). It is worth bearing in mind that all patients are similarly affected by dyslipidemia; therefore, it is reasonable to infer that the presence of T2D in the G1 + G2 groups could have an additive effect for the higher expression of *SOS1* in comparison to the G3 normoglycemic patients.

In contrast, the *BRCA2* gene was downregulated in G1 + G2 compared to G3. This result is interesting because this gene is related to the repair of the damage to the double-stranded DNA pathway indicating that T2D patients may have a deficiency in this process. This is in agreement with Corbi et al. [21], who found higher irreversible damage to the double-stranded DNA in T2D patients than in those without the disease (G3) by utilizing the micronucleus test in the same patients enrolled in the present study.

Considering the well-controlled diabetics (G2) as reference, we found out that the *CCDN3*, *CEBPD*, and *MAP2K5* genes were upregulated in G2 when compared to G1 (poorly controlled diabetics) (Figure 1(b)). Taking into mind, the gene functions are as follows: (i) *CCDN3* gene is associated with the IL-5 immune-related response and with IGF-1 (growth factor similar to insulin-like 1) receptor signaling; (ii) *CEBPD* gene is associated with the IL-6 and IL-17 signaling pathways and; (iii) *MAP2K5* gene is related to T lymphocytes and the inflammatory response; it seems to indicate that the well-controlled diabetics (G2) could better orchestrate the immune system functions. Also, we found an overexpression of the enolase 2 (*ENO2*, alias *NSE)* and vesicle-associated membrane protein 2 (*VAMP2)* genes in G2 subjects. The function of the *VAMP2* gene is linked to the secretion of insulin by pancreatic *β*-cells. The VAMP-2 protein is also associated with the regulation of GLUT-4 trafficking and fusion in adipocytes. In animals with diabetes induced by streptozotocin and nicotinamide, resveratrol supplementation increased the *VAMP2* gene expression and blood insulin level, as well as reduced the fasting blood glucose and improved the insulin resistance [26]. Because we validated here higher expression of the *VAMP2* gene in the G2 group, it seems to indicate that the good glycemic control contributes to increase the insulin production in these patients, in comparison to G1 poorly controlled T2D patients. Obviously, it is essential to T2D patients to achieve and maintain the glycemic control, preferentially without adding basal insulin therapy, because it was related to increased number of patients experiencing hypoglycemia during 1 year of follow-up of basal insulin therapy [27]. Interestingly, intensive glycemic control was associated with higher rates of severe hypoglycemia than less intensive control, as raised by the ACCORD (Action to Control Cardiovascular Risk in Diabetes), ADVANCE (Action in Diabetes and Vascular Disease: Preterax and Diamicron MR Controlled Evaluation), and VADT (Veterans Affairs Diabetes Trial) studies [28–30].

Another DEG in the G2 versus G1 comparison is the *ENO2*, which encodes an intracellular protein present mainly in the neuronal cytoplasm, in central and peripheral neuroendocrine cells [31, 32], and in white blood cells (http://www.genecards.org/cgi-bin/carddisp.pl?gene=ENO2&keywords=ENO2). Interestingly, *ENO2* is also associated with the glycolysis and gluconeogenesis processes (http://lsresearch.thomsonreuters.com/maps/9300). *ENO2* or *NSE* is readily secreted into the cerebrospinal fluid and blood after tissue injury and is related to diabetic retinopathy [31, 33]. Retinopathy, neuropathy, and nephropathy are common complications in patients with diabetes mellitus. Early diabetic retinopathy includes a neurodegenerative component, and diabetic retinopathy is generally viewed as a neurovascular disease [33]. Circulating *ENO2*/*NSE* mRNA levels were lower in diabetics with neuropathy (mean HbA1c = 8.2%) than in those without neuropathy (mean HbA1c = 6.6%) [31]. Here, we also found significant lower levels of circulating *ENO2* mRNA levels in G1 (mean HbA1c = 10.4%, poorly controlled T2D) than in G2 (mean HbA1c = 6.6%, well-controlled T2D) (Figure 1(b)). We noticed from the 30 patients in each group that retinopathy affected 6 patients in the G1 and 1 patient in the G2. Retinopathy was also related to the poor glycemic control of T2D [34].

Still comparing G2 versus G1, the *LGALS12* (lectin, galactoside-binding, soluble 12) gene was upregulated in G1. Genome-wide association studies (GWAS) available at: http://www.gwascentral.org/study accessed (Aug. 24, 2016) demonstrated the *LGALS12* gene as risk loci of BMI (HGVST308) and is associated with glycemic traits (HGVST463). In spite of the validation of *PPAP2B* and *AGBL5* genes, demonstrated *p* values are not significant after Bonferroni's correction, and if we consider that it is not necessary to correct for multiple tests to obtain *p* values of qPCR analysis because each gene was independently investigated, we can assume that these genes were also validated. Whether we consider this, our results are in line with GWAS studies which associated *PPAP2B* gene with T2D (HGVST3; HGVST5), glycated hemoglobin levels (HGVST618), and glycemic traits (HGVST463), while the *AGBL5* was associated with GWAS of glycemic traits (HGVST463).

When comparing the well-controlled T2D (G2) patients versus normoglycemics (G3), besides the aforementioned *HLA-DQB1* gene, we found that *SEC13* and *MCM4* genes presented downregulation in both microarray and RT-qPCR. Interestingly, *SEC13* and *MCM4* genes were also associated with GWAS of glycemic traits (HGVST463) and glycated hemoglobin (HGVST618). *MCM4* participated in the DNA replication in the early S phase, and altered expression and methylation of *MCM4* gene were recently observed in the zebrafish model of metabolic memory (MM). This was defined as the persistence of diabetic (DM) complications even after glycemic control was pharmacologically achieved [35]. According to Leontovich et al., the results were consistent with human diabetic epigenetic studies and provided one explanation for the persistence of long-term tissue complications as seen in diabetes [35]. Furthermore, in agreement with the lower levels of *MCM4* gene in the PBMC of well-controlled T2D (G2) patients (Figure 1(c)), decreased expression of *MCM4*, leading to decreased DNA replication of vascular smooth muscle cells, was demonstrated [36]. This occurs by the overexpression of PGC-1*β*, which is upregulated by metformin. Therefore, a functional role of *MCM4* gene was presented in the context of vascular injury commonly found in diabetes [36].

Lastly, comparing G1 versus G3, we found an upregulation of the *PPIC* gene in G1. *PPIC* gene was associated with endometrial, kidney, and lung neoplasms. Some studies have demonstrated an increase of malignant neoplasms in T2D patients [37, 38]. In addition, the *PPIC* gene was associated with GWAS of glycemic traits (HGVST463) and glycated hemoglobin (HGVST618).

By using the candidate genes/RT-qPCR approach in a well-evaluated cohort, we presented some DEGs in well- and poorly controlled T2D patients. Although our findings are relevant in the context of T2D, it is important to discuss that the present study has some limitations. We agree that there is a lack of a longitudinal study of data tracking of diseases in our patients, and the blood analysis was made in a unique time point, making it difficult and harming the best diagnosis of a T2D patient as well as the poorly controlled. In the present study, we did not assess the genes at the translational level; that is, we did not quantify the proteins encoded by the genes investigated here. Certainly, further studies could clinically follow up the patients enrolled here and could focus on the translational levels of the genes investigated in the present study. Proteomics studies could ascertain the present mRNA findings or reveal different results, since the gene regulation comprises very complex processes. Because the expression profiling was investigated in PBMC, we cannot exclude the possibility that gene expression could be influenced by proportions of the blood cell types in the circulation, as inferred by Miranda et al. [39]. However, we assessed the amount of lymphocytes, neutrophils, eosinophils, and monocytes in each patient investigated here and they were statistically similar among groups, demonstrating that our findings were not influenced by a specific cell type.

Despite the mentioned limitations, this study brings functional information related to glycemic control on gene expression, which was related to the immune response and regulation of glycemic and lipid metabolism, as well as DNA replication and damage. Even though the present study is not the first evidence of the majority association of genes investigated here with T2D, they could be thought as more newness in the association with glycemic metabolism than others, such as the HLA-DQA1, HLA-DQB1, *VAMP2*, and *ENO* genes.

We concluded that some candidate genes potentially associated with adequate glycemic control in T2D patients were validated. Moreover, this study contributed to the knowledge of how the glycemic control could systemically influence gene expression. Further case-control studies and meta-analysis approach with strict clinical selection of larger and ethnic diverse populations should be made to confirm and strengthen our results.

## Figures and Tables

**Table 1:** Characteristics of the sample: demographic, physical, biochemical, and diabetic data (mean ± SD).

	Group 1 *n* = 30	Group 2 *n* = 30	Group 3 *n* = 30
Gender (F/M)	18/12	20/10	17/13
Age (mean ± SD)	48.0 (±7.6)	50.3 (±6.7)	49.0 (±7.5)
Ethnicity (white/brown/black)	13/10/7	15/11/4	20/8/2
BMI (m/kg^2^)	30.5 (±5.2)	31.4 (±4.1)	28.4 (±3.8)
Abdominal circumference (cm)^∗^	104.3 (±14.6)	109.3 (±10.8)^a^	98.1 (±9.9)
Waist/hip proportion	1.0 (±0.1)	1.0 (±0.1)	0.9 (±0.1)
Fasting glucose (mg/dl)^∗^	226.6 (±74.2)^a,b^	137.5 (±41.4)^a^	90.0 (±6.4)
HbA1c (%)^∗^	10.4 (±1.9)^a,b^	6.6 (±0.9)^a^	5.4 (±0.6)
Insulin (U/l)^∗^	19.7 (±20.9)^a^	21.1 (±21.5)^a^	12.6 (±8.5)
Time since DM onset (years) (mean ± SD)	6.2 (±4.2)	5.2 (±6.6)	—
Presence of diabetes			
Total complications^c^	19^c^	12	—
Medication for DM control			
Hypoglycemic	20	15	—
Insulin	1	1	—
Hypoglycemic/insulin	8	5	—
None	1	9	—

SD = standard deviation. ^∗^*α* = 0.017 (since Bonferroni's correction = 0.05/3, i.e., three comparisons); ^a^significant *p* value in relation to group 3; ^b^significant *p* value in relation to group 2 (Kruskal-Wallis test, Dunn's posttest); ^c^*p* < 0.05 in relation to group 2, to access differences regarding complications of diabetes (Mann–Whitney test, *α* = 5%); some data from G1, G2, and G3 were also presented in de Souza Bastos et al. [19], Bastos et al. [20], and Corbi et al. [21].

**Table 2:** DEGs detected by microarray chosen as good candidates for validation by RT-qPCR.

Comparison	Gene title	Gene symbol	Microarray FC (FDR *p* value)	RT-qPCR *p* value (validation)
*G1 + G2 versus G3*				
^@^213831_at #Hs03007426 mH	Major histocompatibility complex, class II, DQ alpha 1	*HLA-DQA1*	127.80 (0.002)	<0.0001
^@^212777_at #Hs00893134_m1	Son of sevenless homolog 1 (drosophila)	*SOS1*	2.60 (0.005)	0.005
^@^222381_at #Hs00918237_m1	Aryl-hidrocarbon receptor repressor	*PDCD6*	−2.43 (0.007)	NV
^@^208368_s_at #Hs00609073_m1	Breast cancer 2, early onset	*BRCA2*	−1.46 (0.009)	0.008
^@^210808_s_at #Hs01071081_m1	NADPH oxidase 1	*NOX1*	−1.43 (0.002)	NV

*G2 versus G1*				
^@^231857_s_at #Hs01005454_g1	ATP/GTP-binding protein-like 5	*AGBL5*	6.08 (0.002)	0.042
^@^1562028_at #Hs00236949_m1	Cyclin D3	*CCDN3*	1.99 (0.006)	0.0003
^@^213006_at #Hs00270931_s1	CCAAT/enhancer-binding protein (C/EBP), delta	*CEBPD*	1.89 (0.007)	<0.0001
^@^214792_x_at #Hs00360269_m1	Vesicle-associated membrane protein 2	*VAMP2*	1.67 (0.001)	0.0001
^@^201313_at #Hs01102367_g1	Enolase 2 (gamma, neuronal)	*ENO2*	1.66 (0.0008)	<0.0001
^@^211370_s_at #Hs00177134_m1	Mitogen-activated protein kinase 5	*MAP2K5*	1.39 (0.001)	0.027
^@^212226_s_at #Hs00170359_m1	Phosphatidic acid phosphatase type 2B	*PPAP2B*	−2.68 (0.005)	0.021
^@^223828_s_at #Hs00263821_m1	Lectin, galactoside-binding, soluble 12	*LGALS12*	−2.09 (0.007)	0.0004

*G2 versus G3*				
^@^209480_at #Hs03054971_m1	Major histocompatibility complex, class II, DQ beta 1	*HLA-DQB1*	131.31 (0.005)	<0.0001
^@^211571_s_at #Hs00171642_m1	Versican	*VCAN*	1.73 (0.009)	NV
^@^212142_at #Hs00907398_m1	Minichromosome maintenance	*MCM4*	−4.46 (0.0001)	0.0006
^@^239617_at #Hs01115007_m1	SEC13 homolog (*S. cerevisiae*)	*SEC13*	−2.34 (0.007)	0.008
^@^205672_at #Hs00166045_m1	Xeroderma pigmentosum, complementation group A	*XPA*	−1.29 (0.003)	NV

*G1 versus G3*				
^@^204517_at #Hs00917412_m1	Peptidylprolyl isomerase C (cyclosphilin C)	*PPIC*	2.21 (0.008)	0.001
^@^1554586_a_at #Hs01598095_g1	Rho-related BTB domain containing 2	*RHOBTB2*	2.13(0.007)	NV
^@^1559921_at #Hs00169777_m1	Platelet/endothelial cell adhesion molecule (CD31 antigen)	*PECAM1*	1.86 (0.004)	NV
^@^215723_s_at #Hs00160118_m1	Phospholipase D1, phosphatidylcholine-specific	*PLD1*	−3.77 (0.005)	NV

FC = fold change; FDR = false discovery rate method according the Benjamini-Hochberg procedure was used for correcting each *p* value for multiple hypothesis testing; NV = microarray not validated by RT-qPCR; ^@^ = probe set ID; # = TaqMan code of Applied Biosystems.
